# The promoting effect of exercise motivation on physical fitness in college students: a mediation effect model

**DOI:** 10.1186/s12889-023-17154-w

**Published:** 2023-11-14

**Authors:** Yudu Liu, Xiaobing Zhai, Yanan Zhang, Can Jiang, Jing Zeng, Mei Yang, Xinyan Xie, Feng Zhou, Bing Xiang

**Affiliations:** 1 Research Center for Health Promotion in Women, Youth and Children, Hubei Province Key Laboratory of Occupational Hazard Identification and Control, School of Public Health, Wuhan University of Science and Technology, 430065, Wuhan, Hubei Province China; 2 Center for Artificial Intelligence Driven Drug Discovery, Faculty of Applied Sciences, Macao Polytechnic University, Macao, China; 3 The University of Edinburgh, Edinburgh, UK

**Keywords:** College students, Exercise motivation, Physical fitness, Physical activity mediation effect

## Abstract

**Background:**

In recent years, the physical fitness situation of college students is worrying in China. Exercise motivation is of great importance for the physical activity and physical fitness. However, existing studies have paid limited attention to the specific effect between exercise motivation, physical activity and physical fitness, and even less to the different genders and exercise motivation dimensions. This study aimed to investigate the promoting effect of sport motivation on physical fitness with different gender and dimension in college students.

**Methods:**

Physical fitness levels of 2544 college students in Wuhan and Jingzhou city were measured. Exercise motivation and physical activity was assessed using the Chinese version of the motives for physical activities measure-revised scale and the physical activity survey scale, respectively. Correlation analysis and structural equation model were used to explore the relationship between exercise motivation, physical activity and physical fitness. Bootstrap method was used to test the mediating effect. Multilevel regression analysis was used to examine the effects of different dimensions of exercise motivation on PF.

**Results:**

The exercise motivation of college students was directly related to physical fitness (effect value: 0.307) or indirectly related through the mediating effect of physical activity (effect value: 0.092). The mediation effect percentage of physical activity on exercise motivation and physical fitness in male (51.20%) was greater than female (27.18%), and the standardized regression coefficient of the health dimension to PF was β = 0.151, *P* < 0.001, and the competence dimension to PF was β = 0.189, *P* < 0.001.

**Conclusions:**

The exercise motivation of college students can directly influence PF or indirectly influence PF through the mediating effect of PA. The promoting effect of exercise motivation and PA on PF in college students is related to gender and dimensions of exercise motivation. Therefore, we can improve physical fitness levels of college students by promoting their exercise motivation (especially for health motivation and competence motivation) and increasing their participation in physical activity. This study provides new strategies for improving physical fitness in college students.

**Supplementary Information:**

The online version contains supplementary material available at 10.1186/s12889-023-17154-w.

## Introduction

Physical fitness (PF) is considered as a key factor for physical and mental health [1], and PF is of great importance for the health of college students. It has been reported that better PF may related to a better state of mental-health and health-related quality of life [2]. There were studies also found that increased PF may have important support to the academic performance [3], and lower performance in health-related PF was associated with poorer sleep quality among college students [4]. Although numerous studies have confirmed the importance of PF to college students, the PF situation of college students is still worrying. In recent years, with the continuously increase of the prevalence for obesity and sedentary lifestyle, PF has gradually become a great concern in the health of Chinese college students. According to Chinese *National Physical Health Standards for Students (Revised in 2014)*, the PF for college students were evaluated based on seven aspects: body shape, physical function, speed, flexibility, bounce, strength and endurance. The data from the Chinese nationwide student PF surveillance programme showed that the body mass index (BMI) and prevalence of obesity has been increasing in the Chinese college students, meanwhile the lung capacity is decreasing year by year, and the speed, endurance and bounce of college students are also on the decline. Therefore, it is urgent to explore the affecting factors and promote the PF of college students.

The lack of adequate physical activity (PA) is one of the most dominant factors for the PF decline of college students [5]. PA is defined as any physical movement of skeletal muscle that consumes energy [6]. The new WHO 2020 guidelines on PA recommend an average of 60 min/day of moderate-to-vigorous intensity aerobic PA across the week provides health benefits among children and adolescents [7]. Additionally, in terms of normative data, it appears that healthy adults can take anywhere between approximately 4,000 and 18,000 steps/day, and that 10,000 steps/day is a reasonable target for healthy adults [8]. In 2016, China released the National Fitness Program, which recommends daily 30–60 min moderate PA. However, only roughly one-thirds (34%) of the Chinese college students achieved the PA recommendation [9]. A study among Chinese college students have found that a significant relationship between PA and PF, those students who lack PA have a 1.25 times higher risk of obesity than those who actively participate in PA. Likewise, the probability of failure in the grip strength test and the standing long jump was also higher, with increases of 2.39 fold and 1.39 fold, respectively [5]. Therefore, it is necessary to promote more active participation in PA to improve PF. Previous studies have found that the PA of college students is closely related to the exercise motivation [10], and the motivational factors support a better predisposition towards PA [11], these show that exercise motivation is a critical factor in supporting sustained PA. Thus, it is particularly important to study how to promote exercise motivation to enhance PA participation.

In numerous studies, the relevant achievements of Self-Determination Theory (SDT) are clear with respect to investigation of exercise motivation [12]. In the 1980s, American psychologists Deci and Ryan developed SDT. SDT is a viable conceptual framework to study antecedents and outcomes of motivation for health-related behaviors [12]. The study of exercise motivation is an extension and application of SDT, and a more complete understanding of the dimensions of exercise motivation is key to developing effective PA interventions. Specifically, SDT consist of intrinsic motivation (intrinsic motivation is defined as doing an activity because of its inherent satisfactions) and extrinsic motivation (extrinsic motivation refers to doing an activity for instrumental reasons, or to obtain some outcome separable from the activity) [13]. Highly autonomous intrinsic motivation is more able to control one's behaviour to achieve good results compared to extrinsic motivation [14]. Exercise motivation could be further divided into five dimensions: intrinsic motivation includes fun, competence and appearance, and extrinsic motivation includes the health and social [10]. Previous studies have showed that the exercise volume and aerobic fitness levels of college students could be improved by motivating their exercise participation [15], and the highly correlation between exercise motivation for PA participation and PF in the college students has been verified, which serves as a theoretical basis for the establishment of a conceptual model of intermediary effects. Therefore, PA is an indispensable intermediate factor when studying the influence of exercise motivation of college students on PF. We attempted to develop a mediation effect model that promotes the PF and PA of college students. Exercise motivation of students and PF are used as the antecedent and outcome variables, respectively. We also considered the mediating effects of PA to investigate the relationship between exercise motivation, PA, and PF. In this study, we conducted a cross-sectional study in students from two universities in Wuhan and Jingzhou city, and based on data obtained for exercise motivation, PA and PF, we aimed to explore the correlation between the three through structural equation model.

**Fig. 1:**
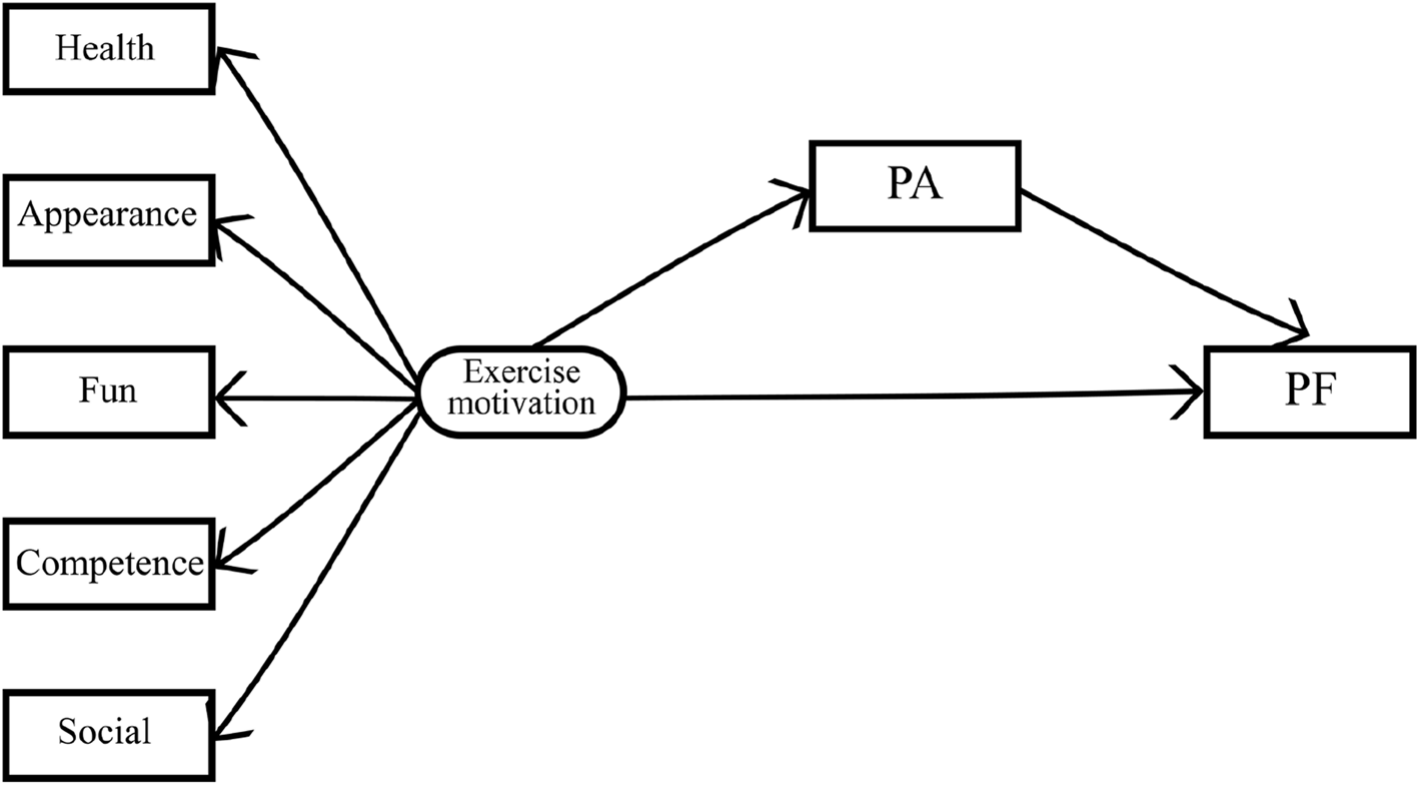
Conceptual framework

There is evidence suggesting that potentially important gender differences in exercise motivation for physical activity. Male reported higher levels of exercise motivation than did female for challenge, competition, social recognition, and strength and endurance [16]. It is of note that also differences between male and female in PF and PA, which may be due to their different exercise motivation for participating in physical activity [17]. Obviously, gender is a variable that must be considered in this study. Furthermore, Scientists agree that understanding the dimensions of exercise motivation of individuals to participate in PA is of great practical value [18]. Adopting such a perspective, in a study of school being introduced to a new physical activity, found that a health-focussed message was in most respects more effective than a control condition, whereas an appearance-focussed message was in most respects less effective. It is suggested that an intervention will be most effective if its content is varied so as to appeal to the different exercise motivation of individuals [19]. Thus, is the effect of exercise motivation on PF in college students related to dimensions of exercise motivation? Which is also need to be addressed. However, existing studies have paid limited attention to the different genders of exercise motivation of college students, and even less to the relationships among their dimension of exercise motivation for PF. Therefore, we also aimed to further explore the effects of exercise motivation on PF among college students of different genders, and the effects of different dimensions of exercise motivation on PF. Our research hypotheses (H1-H4 see Fig. 1) were as follows:

- H1: Exercise motivation can significantly predict PF.
- H2: Exercise motivation can indirectly promote PF through an intermediary of PA.
- H3: The effect of exercise motivation on PF in college students is related to gender.
- H4: The effect of exercise motivation on PF in college students is related to dimensions of exercise motivation.

Based on these hypotheses and questions, this study aimed to investigate methods to promote PA and PF among college students based on the SDT. The ultimate goal is to promote exercise motivation of college students, raise their awareness of PA, improve their PF and reduce the prevalence of chronic diseases.

## Methods

### Participants

This study conducts two-stage stratified random sampling. In the first stage, the 17 urban areas in Hubei Province are divided into 2 layers according to the economic level (high and medium/low), and the two universities were randomly selected in two different layers of cities. In the second stage, students were randomly selected according to the proportion over the each grade students in university. Questionnaires and PF tests were conducted on 3734 students at two universities in Wuhan and Jingzhou city, Hubei province between October and November 2020. Before formal investigation and testing, the informed consent of the subjects involved were obtained in this study. Inclusion criteria: ① Students aged between 18 and 24 years old (*N* = 3498). ② Students volunteered to participate in this study. Exclusion criteria: ① Students with incomplete data of PF test (*N* = 823). ② Dropped out of the study (*N* = 114). ③ Students with contraindications to exercise (e.g., hypertension, asthma and acute inflammation, *N* = 17). Finally, 2544 college students were included, mean age was 19.27 ± 1.19 years. In total, 1324 (52.04%) were males and 1220 (47.96%) females; 1803 (70.90%) were freshman/sophomore and 741 (29.10%) junior/senior. Sample selection flowchart see Supplementary Fig. 1.

### Evaluation instruments

#### Exercise motivation scale

The motives for physical activities measure‑revised (MPAM‑R) compiled by Ryan is a commonly used tool for determining exercise motivation [10]. To simply measure exercise motivation, Chen et al. reduced the Chinese version of the MPAM-R [20]. In this study, a simplified version of the motivation scale was used, which includes five exercise motivation dimensions, namely, health, appearance, fun, competence and social, with a 15-item simplified scale on a Like5 scale, each exercise motivation dimension includes 3-item, with motivation rated from "none" to "very strong" on a scale of 1–5, with higher scores indicating higher levels of motivation. The Chinese version of the scale is adequately reliable and valid in this study (Cronbach’s ɑ = 0.933, KMO = 0.929), and the reliability coefficients of each sub-scale ranged from 0.767 to 0.896.

#### Physical activity scale

The physical activity survey scale designed by Zhang Yuci was used. The scale is designed for Chinese college students based on Borg's Rating of Perceived Exertion (RPE) [21] and the previous research results of Chinese researcher. This scale of four questions was used to investigate the PA behaviour of university students. Question 1:"How many times a week do you attend PA after school?"; Question 2: "How long in total do you attend PA each week after school?"; Question 3: "What are the characteristics of each PA attendance?"; Question 4: "What is the PA program you attend most often outside of school hours? What is the average length of time you attend PA each time?". Question 1 uses an eight-level scoring (1 = less than once to 8 = every day), question 2 uses a nine-level scoring (1 = none to 9 = 7 h or longer), question 3 uses a four-level scoring (1 = no sensation or slight warmth to 4 = sweating and shortness of breath), question 4 uses a six-level scoring (1 = I have no plans for physical exercise to 6 = I have been exercising regularly and for over 3 years), with the total score being from 4 to 27. A higher score in the physical activity survey scale indicates a higher PA behaviors level of college students. The physical activity survey scale is adequately reliable and valid in this study (Cronbach’s ɑ = 0.868, KMO = 0.837).

#### Physical fitness evalauation

The PF test was conducted in accordance with the relevant requirements of *The National Student Physical Fitness Score* issued by China, the items of the PF test were BMI, lung capacity, 50-m run, seated forward bend, standing long jump, pull-up (male)/sit-up (female) and 1,000-m run (male) / 800-m run (female). Each tests result is converted into a percentage score. It classifies students into four levels of PF status. The level standards were as follows: excellent standard ≥ 90 points, good standard = 80–89 points, passing standard = 60 – 79 points, not passing standard < 60 points. PF score = BMI * 15% + lung capacity * 15% + 50-m run * 20% + seated forward bend * 10% + standing long jump * 10% + pull-up (male)/sit-up (female) * 10% + 1,000-m run (male) / 800-m run (female) * 20%. The PF test item weights see Supplementary Table 1. During the PF test, the students completed a questionnaire. Prior to the questionnaire, standardized training was provided to the investigators. In order to ensure the accuracy and scientific nature of the PF test methods for college students, all items of the PF test are conducted by a full-time teacher specialising in physical education. In addition, to reduce unnecessary errors each test element is monitored by the same test teacher.

### Statistical analysis

The IBM SPSS Statistics 26.0 was used for data processing, Amos24.0 was used to establish and analyze the structural equation model among variables. *x*^*2*^/df, GFI, AGFI, CFI and RMSEA were used to evaluate the goodness of the model-data fit [22]. Population characteristics is statistically described using frequency (N), mean (M) and standard deviation (SD). For data with normal distribution, comparisons between two groups were performed with t-test for continuous variables. Correlation analysis was conducted to investigate the correlation among exercise motivation, PA, and PF. Bootstrap method was used to test the mediating effect. Multilevel regression analysis was used to examine the effects of different dimensions of exercise motivation on PF. A two-side a less than 0.05 was considered to define statistical significance.

## Results

### Descriptive statistics and correlation analysis

Table 1 showed that mean scores of the exercise motivation (50.73 ± 11.31). Health motivation (11.22 ± 2.43) were highest in the five dimensions of exercise motivation, followed by appearance motivation (10.44 ± 2.85), fun motivation (10.32 ± 2.50), competence motivation (9.43 ± 2.88), and social motivation (9.32 ± 2.97). In the comparison between male and female, male had higher mean scores for health motivation, fun motivation, competence motivation and social motivation, and female had higher mean scores for appearance motivation. And male had higher mean PA scores and lower mean PF scores than female. In addition, the results of the correlation analysis in Table 2 showed that exercise motivation was significantly related to PA and PF (*P* < 0.05); Moreover, a high degree of correlation was found among five dimensions of exercise motivation with PA and PF (*P* < 0.05).

**Table 1:** Survey results of scores of exercise motivation, PF and PA

	Total *N* = 2544	Male *n* = 1324(52.04%)	Female *n* = 1220(47.96%)	*P* (male and female)
Exercise motivation	50.73 ± 11.31	52.02 ± 11.38	49.33 ± 11.09	< 0.001
Health	11.22 ± 2.43	11.51 ± 2.44	10.91 ± 2.40	< 0.001
Appearance	10.44 ± 2.85	10.22 ± 2.87	10.69 ± 2.82	< 0.001
Fun	10.32 ± 2.50	10.60 ± 2.45	10.02 ± 2.52	< 0.001
Competence	9.43 ± 2.88	9.89 ± 2.90	8.93 ± 2.76	< 0.001
Social	9.32 ± 2.97	9.81 ± 2.92	8.79 ± 2.92	< 0.001
PA	12.91 ± 3.15	13.86 ± 3.22	11.88 ± 2.72	< 0.001
PF	72.51 ± 7.95	70.20 ± 8.50	75.02 ± 6.41	< 0.001

**Table 2:** Correlation analysis of exercise motivation, five dimensions of exercise motivation, PF and PA

	Exercise motivation	Health	Appearance	Fun	Competence	Social	PA	PF
Exercise motivation	1							
Health	0.814**	1						
Appearance	0.708**	0.528**	1					
Fun	0.900**	0.749**	0.542**	1				
Competence	0.873**	0.605**	0.435**	0.746**	1			
Social	0.859**	0.559**	0.427**	0.731**	0.816**	1		
PA	0.431**	0.393**	0.232**	0.393**	0.408**	0.369**	1	
PF	0.119**	0.141**	0.061**	0.101**	0.129**	0.068**	0.106**	1

^*^P < 0.05

^**^P < 0.01

### Bootstrap analysis in total, male and female

Based on the results of the correlation analysis. A Structural equation model for exercise motivation, PA and PF were constructed considering the relationship of outcome variable (PF) and antecedent variables (exercise motivation and PA). The final well-fitting model was obtained by progressively modifying the path (see Fig. 1). The model fit indicators are reported in Supplementary Table 2. Table 3 showed that the specific effect values and 95% CI of each path in this model. Therefore, the results validate hypothesis H1 and H2 of this study, and the results showed that exercise motivation positively affected PF. 95% CI demonstrated that PA partially mediates between exercise motivation and PA.

**Table 3:** Bootstrap analysis of the mediation effect size and significance test of PA in exercise motivation and PF

	**Path**	**Effect**	**SE**	**Z-value**	**95%CI**	***P***
**Total**	Exercise Motivation → PF (Direct effect)	0.307***	0.079	3.886	[0.153, 0.457]	< 0.001
	Exercise Motivation → PA → PF (Mediation effect)	0.092**	0.034	2.706	[0.026, 0.159]	0.007
	Exercise Motivation → PF (Total effect)	0.399***	0.070	5.700	[0.261, 0.536]	< 0.001
**Male**	Exercise Motivation → PF (Direct effect)	0.345**	0.105	3.286	[0.140, 0.556]	0.001
	Exercise Motivation → PA → PF (Mediation effect)	0.362***	0.055	6.582	[0.261, 0.476]	< 0.001
	Exercise Motivation → PF (Total effect)	0.707***	0.093	7.602	[0.532, 0.900]	< 0.001
**Female**	Exercise Motivation → PF (Direct effect)	0.285**	0.089	3.167	[0.110, 0.461]	0.002
	Exercise Motivation → PA → PF (Mediation effect)	0.106**	0.034	3.118	[0.042, 0.175]	0.002
	Exercise Motivation → PF (Total effect)	0.390***	0.083	4.699	[0.231, 0.549]	< 0.001

^*^P < 0.05

^**^P < 0.01

^***^P < 0.001

In the total participants, the direct and total effect of exercise motivation on PF were 0.523 and 0.823, respectively. PA played a partial mediation effect between exercise motivation and PF, and the mediation effect was 0.300, which was accounted for 36.45% of the total effect; In the male group, the direct and total effect of exercise motivation on PF were 0.345 and 0.707, respectively. PA played a partial mediation effect between exercise motivation and PF, and the mediation effect was 0.362, which was accounted for 51.20% of the total effect; In the female group, the direct and total effect of exercise motivation on PF were 0.285 and 0.390, respectively. PA played a partial mediation effect between exercise motivation and PF, and the mediation effect was 0.106, which was accounted for 27.18% of the total effect (Table 3). The above results demonstrated that the mediation effect percentage was significantly higher in male than in female. The results validate hypothesis H3 of this study.

### Hierarchical multiple regression analysis of PF

The PF was taken as the dependent variable, the demographic characteristics, PA and five dimensions of exercise motivation (health, appearance, fun, competence and social) were taken as independent variables for the hierarchical regression analysis. The first layer was the three demographic characteristics, the second layer was the PA, and the five dimensions of exercise motivation were included in the third layer. Table 4 showed that the regression equation is statistically significant and explains 16.4% of the total variation in PF after controlling for gender, age, grade, and PA. The standardized regression coefficient of the health dimension to PF was β = 0.151, *P* < 0.001, and the competence dimension to PF was β = 0.189, *P* < 0.001. The standardized regression coefficient of the appearance dimension to PF was β = -0.108, *P* < 0.001, and the social dimension to PF was β = -0.075, *P* < 0.05. The results validate hypothesis H4 of this study.

**Table 4:** Hierarchical multiple regression analysis of PF

Layers	Variables	β	t	R^2^	Adj R^2^	ΔR^2^	F
Step 1	Gender	0.307***	15.971	0.093	0.092	0.093	86.375***
	Age	0.034	1.289				
	Grade	-0.025	-0.937				
Step 2	Gender	0.375***	19.116	0.138	0.137	0.046	135.029***
	Age	0.036	1.380				
	Grade	0.001	0.047				
	PA	0.227***	11.620				
Step 3	Gender	0.396***	20.051	0.167	0.164	0.029	17.643***
	Age	0.029	1.136				
	Grade	0.010	0.404				
	PA	0.175***	8.252				
	Health	0.151***	5.296				
	Appearance	-0.108***	-4.795				
	Fun	-0.062	-1.784				
	Competence	0.189***	5.494				
	Social	-0.075*	-2.239				

^*^P < 0.05

^**^P < 0.01

^***^P < 0.001

## Discussion

This study explored the relationship between exercise motivation, PA and PF among college students through a cross-sectional design using structural equation model. The results of this study showed a significant positive correlation between exercise motivation, PA and PF in college students. exercise motivation of college students can influence PF directly or indirectly through the mediating effect of PA. This study can prove that the greater exercise motivation and the better participation in PA could promote the PF status. This study is an extension and application of exercise motivation theory based on STD.

### Extension and application of exercise motivation theory based on STD

One of the core objectives of the exercise motivation theory based on STD is to stimulate and encourage more people to participation in PA [23]. The results of this study validate the hypothesis H1-H4 of this study, exercise motivation can significantly predict exercise behavior, which suggests that the amount of PA can be increased by increasing the exercise motivation among college students, followed by an increase in the PF. STD consists of intrinsic and extrinsic motivation. This study found that males and females have different motivations to participate in PA, and that different dimensions of exercise motivation have different effect to PF. Our study is an extension and application of exercise motivation theory based on STD.

### Gender differences in different dimensions of exercise motivation among college students

The descriptive analysis results of the different dimensions of exercise motivation scores showed that the highest intensity of exercise motivation of college students was health motivation, followed by appearance motivation, fun motivation, competence motivation and social motivation. This indicated that the primary motivation for college students to participate in PA was to maintain physical health, which is similar to the previous study [24]. The lowest intensity of social motivation suggested that college students do not participate in PA for social reasons to a large extent. In comparison, the external motivation of college students to participate in PA was stronger than the internal motivation. A possible reason for this is that highly autonomous intrinsic motivation is more able to control one's behaviour to achieve good results compared to extrinsic motivation [14]. In addition, male show stronger exercise motivation to participate in PA compared to female, and a study comparing the exercise motives for male and female Mexican college students participate in PA also confirm this [25]. Of the five dimensions of exercise motivation, male have higher mean scores than female in health motivation, fun motivation, competence motivation and social motivation, and female have higher mean scores than male in appearance motivation only. It may suggest that female is more motivated by extrinsic factors to participate in PA, such as weight control and improved appearance, while male is more motivated by intrinsic factors to participate in PA, such as power competition and challenges [16, 26, 27]. This suggests that when designing intervention to potentiate improvement on exercise motivation, the variable gender should be considered.

### PF levels of college students were far from excellent standard, with female overall higher than male

The average PF score (M = 72.51) of college students in this study were far from the Chinese national excellent standard (≥ 90), which indicates that their PF level needs to be further improved, this reminds us that attention should be draw on the strengthening the PF for college students. Therefore, we suggest that universities should actively conduct health promotion activities to promote the exercise motivation of university students and improve their PF. In addition, a study found that there are differences between female and male in relation to the weekly hours of exercise and physical condition, which is higher among male [28]. However, we found an interesting phenomenon that male had higher average PA scores than female, but lower PF scores than female, this is different from previous studies. We speculate that this may be due to the fact that female have better dietary and lifestyle habits, which can have a positive impact on PF, as has been demonstrated in previous studies [29].

### The effects of exercise motivation on PF in college students is related to dimensions of exercise motivation

The results of the hierarchical multiple regression analysis found that five dimensions of exercise motivation (health, appearance, fun, competence and social) predicted PF differently in college students after controlling for gender, age, grade and PA. Our research has confirmed that exercise motivation predicts PF. However, appearance motivation and social motivation are negative factors of predicting PF. It may be that appearance motivation and social motivation can also lead to anxiety, stress, and even depression when people lack of the social status and recognition from appearance, which often causes the person to stop exercising [30, 31]. In contrast, among the five dimensions of exercise motivation, health motivation and competence motivation were the significant positive factors of predicting PF. It has been reported that exercise motivation, such as enjoyment of healthy exercise, feeling competent, have a positive effect on exercise endurance [32], which is an important indicator of PF. In addition, health motivation increased identified regulation, and was positively related to exercise participation, which also promotes PF [33].

This study has some practical applications that are worth highlighting. To enhance PF, we need to consider that university can take measures to promote exercise motivation (especially for health motivation and competence motivation) of students. Our study suggests that not only gender but also dimensions of exercise motivation should be considered when designing interventions to increase exercise motivation to improve PA and PF.

### Limitations

First, our study is cross-sectional, thus causal inferences can not be determined. In the future, we should design longitudinal data collection strategies and methods on the basis of this research, and analyze the strength and direction of the causal relationship of exercise motivation, PA, and PF. Second, this study was conducted on college students at two universities only and it is hoped that the sample population can be expanded in the future for a comprehensive study. Finally, this study lacks data of important factors such as diet and sleep. These factors are associated with PF and they will be collected in our future studies.

## Conclusions

The exercise motivation of college students can directly influence PF or indirectly influence PF through the mediating effect of PA. Health motivation is the primary exercise motivation for college students to participate in PA, and the effects of exercise motivation and PA on PF in college students is related to gender and dimensions of exercise motivation. Based on the corresponding evidence from this study, we can achieve the goal of improving PF levels and reducing the prevalence of chronic diseases among college students by promoting their exercise motivation (especially for health motivation and competence motivation) and increasing their participation in PA, and the gender and exercise motivation should be considered when designing intervention to promote PF. This study provides new strategies for improving PF in college students and new ideas for further researches.

### Supplementary Information

**Additional file 1: Supplementary Fig 1.** Sample selection flowchart

**Additional file 2: Supplementary Table 1**. PF test item weights.

**Additional file 3: Supplementary Table 2.** Model fit indicators.

## Data Availability

The datasets used and/or analyzed during this survey are available from the corresponding author upon reasonable request.

## Abbreviation

PF: Physical fitness
PA: Physical activity
BMI: Body mass index
SDT: Self-determination theory
RPE: Rating of Perceived Exertion
MPAM-R: Motives for Physical Activities Measure-Revised
