# Supplementary figures and images for: The promoting effect of exercise motivation on physical fitness in college students: a mediation effect model
Source: BMC Public Health. 2023 Nov 14;23:2244. doi: 10.1186/s12889-023-17154-w (PMC10644452; doi:10.1186/s12889-023-17154-w)

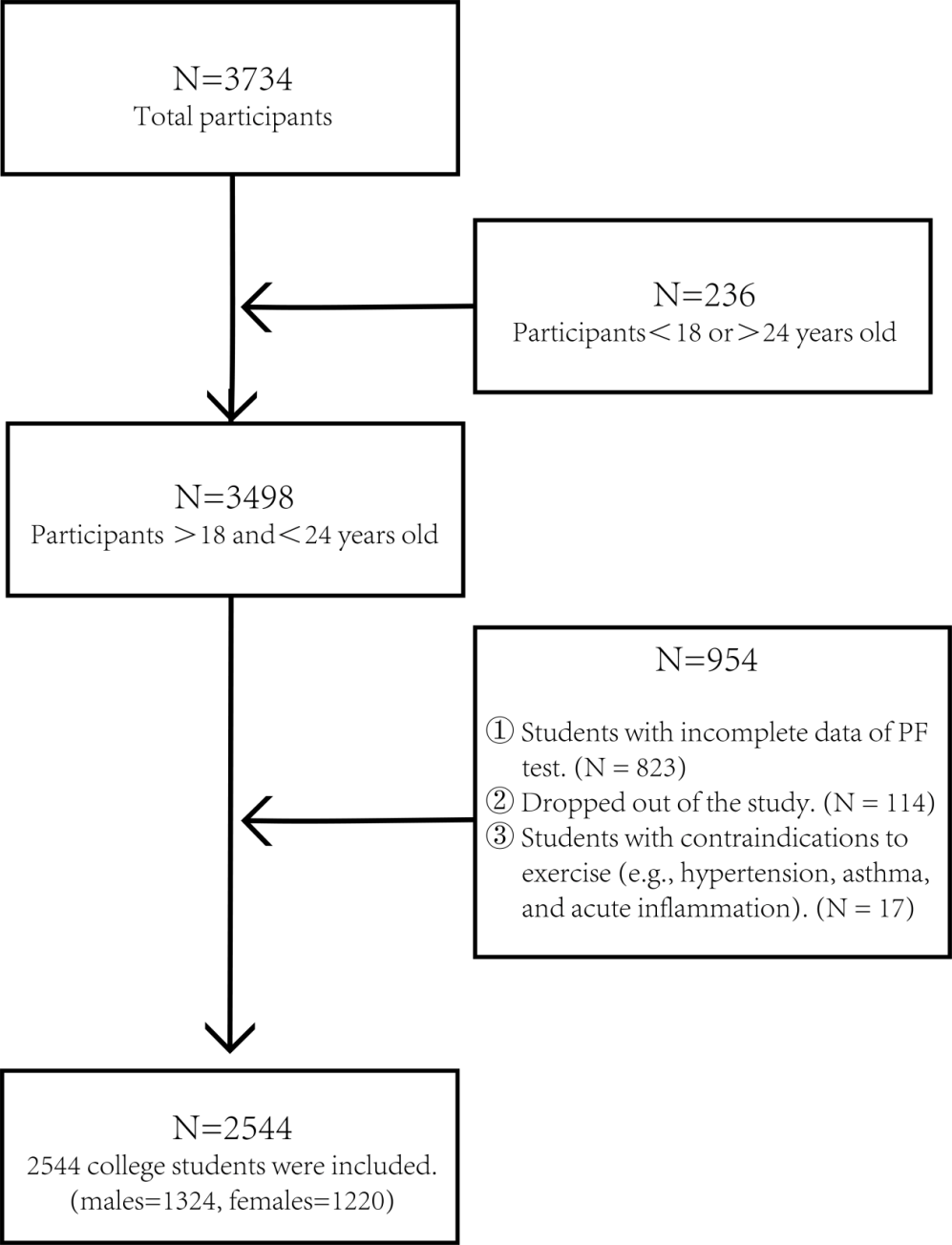


**Supplementary Fig 1.** Sample selection flowchart.

Supplement: Supplementary file 1 — Additional file 1: Supplementary Fig 1. Sample selection flowchart [file 12889_2023_17154_MOESM1_ESM.docx]
