# Supplementary material for: The promoting effect of exercise motivation on physical fitness in college students: a mediation effect model
Source: BMC Public Health. 2023 Nov 14;23:2244. doi: 10.1186/s12889-023-17154-w (PMC10644452; doi:10.1186/s12889-023-17154-w)
Supplement: Supplementary file 2 — Additional file 2: Supplementary Table 1. PF test item weights. [file 12889_2023_17154_MOESM2_ESM.docx]

**Supplementary Table 1**. PF test item weights.

| **PF test items** | **Weights (％)** |
| --- | --- |
| BMI | 15 |
| lung capacity | 15 |
| 50-meter run | 20 |
| seated forward bend | 10 |
| standing long jump | 10 |
| pull-up (male)/sit-up (female) | 10 |
| 1,000-meter run (male) / 800-meter run (female) | 20 |
