# Supplementary material for: The promoting effect of exercise motivation on physical fitness in college students: a mediation effect model
Source: BMC Public Health. 2023 Nov 14;23:2244. doi: 10.1186/s12889-023-17154-w (PMC10644452; doi:10.1186/s12889-023-17154-w)
Supplement: Supplementary file 3 — Additional file 3: Supplementary Table 2. Model fit indicators. [file 12889_2023_17154_MOESM3_ESM.docx]

**Supplementary Table 2.** Model fit indicators.

|  | **χ²/df** | **GFI** | **AGFI** | **CFI** | **RMSEA** |
| --- | --- | --- | --- | --- | --- |
| **Total** | 12.175 | 0.973 | 0.945 | 0.969 | 0.068 |
| **Male** | 9.460 | 0.982 | 0.944 | 0.984 | 0.080 |
| **Female** | 3.018 | 0.994 | 0.980 | 0.996 | 0.041 |
